# Is heterostyly rare on oceanic islands?

**DOI:** 10.1093/aobpla/plv087

**Published:** 2015-07-21

**Authors:** Kenta Watanabe, Takashi Sugawara

**Affiliations:** 1 Okinawa College, National Institute of Technology, 905 Henoko, Nago, Okinawa 905-2192, Japan; 2 Makino Herbarium, Graduate School of Science, Tokyo Metropolitan University, 1-1 Minami-Ohsawa, Hachioji, Tokyo 192-0397, Japan

**Keywords:** Dioecy, distyly, heterostyly, monoecy, oceanic islands, Pacific islands, *Psychotria*, reproductive system

## Abstract

Heterostyly has been considered rare or absent on oceanic islands. However, there has been no comprehensive review on this issue. Is heterostyly truly rare on oceanic islands? What makes heterostyly rare on such islands? To answer these questions, we review the reproductive studies on heterostyly on oceanic islands, with special emphasis on the heterostylous genus *Psychotria* in the Pacific Ocean as a model system. Overall, not many reproductive studies have been performed on heterostylous species on oceanic islands. In Hawaiian *Psychotria*, all 11 species are thought to have evolved dioecy from distyly. In the West Pacific, three species on the oceanic Bonin and Lanyu Islands are distylous (*Psychotria homalosperma*, *P. boninensis* and *P. cephalophora*), whereas three species on the continental Ryukyu Islands show various breeding systems, such as distyly (*P. serpens*), dioecy (*P. rubra*) and monoecy (*P. manillensis*). On some other Pacific oceanic islands, possibilities of monomorphy have been reported. For many *Psychotria* species, breeding systems are unknown, although recent studies indicate that heterostylous species may occur on some oceanic islands. A shift from heterostyly to other sexual systems may occur on some oceanic islands. This tendency may also contribute to the rarity of heterostyly, in addition to the difficulty in colonization/autochthonous evolution of heterostylous species on oceanic islands. Further investigation of reproductive systems of *Psychotria* on oceanic islands using robust phylogenetic frameworks would provide new insights into plant reproduction on oceanic islands.

## Abstract (precis)

Heterostyly has been considered rare on oceanic islands. Is heterostyly truly rare on oceanic islands? What makes heterostyly rare on such islands? To answer these questions, we review the reproductive studies of heterostyly on oceanic islands. Overall, not many reproductive studies of heterostyly have been performed on oceanic islands. Shift from heterostyly to other sexual systems may also contribute to rarity of heterostyly, in addition to difficulty of colonization/autochthonous evolution of heterostylous species on oceanic islands. Further investigation of reproductive systems of the heterostylous genus *Psychotria* on oceanic islands would provide new insights on plant reproduction on oceanic islands.

## Introduction

Plant reproductive systems on islands have attracted the attention of many evolutionary biologists (Carlquist 1974; Ehrendorfer 1979; Bawa 1982; Baker and Cox 1984; Barrett *et al*. 1996; Sakai and Weller 1999; Crawford *et al*. 2011). Many general issues in evolutionary biology can be addressed using island plants (Barrett *et al*. 1996). Reproductive systems affect colonization, establishment, and maintenance and diversification of plants on islands (Carlquist 1974; Barrett *et al*. 1996; Crawford *et al*. 2011). Contradictions between strategies selecting for self-fertilization and cross-pollination have been one of the major issues for the reproductive studies of island plants (Barrett *et al*. 1996; Crawford *et al*. 2011). In an earlier study, Baker (1955) proposed that self-compatible, rather than self-incompatible, plants would be favoured in establishment after long-distance dispersal [later formulated as Baker's Law (Stebbins 1957)]; only one self-fertilizer is needed to reproduce after long-distance dispersal, while self-incompatible plants need at least two individuals. The paucity of pollinators on islands also enhances the advantages of self-compatible plants, because self-incompatible plants are more reliant on suitable pollinators to reproduce. Indeed, homomorphic or heteromorphic incompatible species are usually less common on oceanic islands than on continents (Barrett *et al*. 1996; Crawford *et al*. 2011).

In contrast, poor pollinator service resulting in selfing and inbreeding depression can cause the evolution of sexual dimorphism (Barrett *et al*. 1996). In fact, many major oceanic islands have a high proportion of dioecism (Carlquist 1974; Baker and Cox 1984; Sakai *et al*. 1995*b*; Abe 2006). Moreover, many examples of evolution of dimorphism from monomorphic ancestors are known from oceanic islands, such as the Hawaiian Islands (e.g. *Bidens*, *Broussaisia*, *Cyrtandra*, *Hedyotis*, *Neraudia*, *Santalum*, *Schiedea*, *Wikstroemia*; Sakai *et al*. 1995*b*), the Bonin Islands (e.g. *Callicarpa*, Kawakubo 1990; *Dendrocacalia*, Kato and Nagamasu 1995; *Wikstroemia*, Sugawara *et al*. 2004; *Ligustrum*, Tsuneki *et al*. 2011), the Mascarene islands (e.g. *Chassalia*, Pailler *et al*. 1998*a*; *Dombeya*, Humeau *et al*. 1999; Le Péchon *et al.* 2010), the Juan Fernandez Islands (*Robinsonia*, Bernardello *et al*. 2001) and the Canary Islands (*Bencomia, Marcetella*, Helfgott *et al*. 2000; *Withania*, Anderson *et al*. 2006). Many studies of reproductive systems targeting island plants have focused on the occurrence and evolution of dioecism, but only a handful of studies have investigated the occurrence of heterostyly on oceanic islands.

Heterostyly is a genetically controlled floral polymorphism that promotes outbreeding (reviewed in Vuilleumier 1967; Ganders 1979*b*; Barrett 1992; Barrett and Shore 2008). It has attracted the attention of evolutionary researchers since Darwin (Darwin 1877; Weller 2009; Barrett 2010). Heterostyly includes distyly and tristyly, of which distyly is more common. A distylous population comprises two morphs: one is the short-styled morph (S-morph) with short styles and long anthers, and the other is the long-styled morph (L-morph) with long styles and short anthers. These two morphs usually have reciprocal positions in stigma and anther heights, and usually occur in equal numbers within a population. Distylous species usually possess an incompatibility system that prevents self-fertilization and intramorph fertilization (heteromorphic incompatibility). This herkogamous floral dimorphism is generally thought to be controlled by ‘a heterostylous gene’ (Barrett and Shore 2008). In *Primula*, this heterostylous gene is called as a ‘supergene’, which consists of three tightly linked diallelic genes (Charlesworth and Charlesworth 1979*b*; Lewis and Jones 1992).

It is generally thought that heterostyly is rare or absent on remote oceanic islands (Pailler *et al*. 1998*b*), despite the fact that outcrossing is advantageous for avoiding inbreeding depression (Barrett *et al*. 1996). Colonization is likely to be difficult for self-incompatible plants on oceanic islands and *in situ* evolution of heterostyly is almost impossible due to its complex genetic control system. Heterostyly may also be rare because it evolves into other breeding systems such as self-compatibility or dioecy, due to a paucity of suitable pollinators and a small population size on oceanic islands. Indeed, there are several examples of evolution of dioecy or self-fertilization from distyly on islands (e.g. Godley 1979; Beach and Bawa 1980; Pailler *et al*. 1998*a*). However, there has been no comprehensive review on this issue.

Given this background, we hypothesized that (i) heterostyly is truly rare on remote oceanic islands and (ii) this rarity is partly because of the tendency to shift from heterostyly to dioecism or self-fertilization on oceanic islands in addition to the difficulty in colonization/autochthonous evolution of heterostyly. One of the best ways to test these hypotheses is to compare reproductive systems of lineages distributed on oceanic and continental islands, and on continents using robust phylogenetic hypothesis. First, we briefly summarize the distribution of heterostylous plants on oceanic islands. Second, we document the breeding systems of the distylous genus *Psychotria* occurring on the Pacific islands as a model system for the study of heterostyly on islands. Finally, we discuss the reasons concerning the rarity and evolutionary modification of heterostyly on islands.

In this review, we focus on heterostyly on oceanic islands. We use the term ‘oceanic islands’ following Whittaker and Fernández-Palacios (2007) as ‘oceanic islands are those that have formed over oceanic plates and have never been connected to any continental landmass’. For this reason, the flora and fauna on oceanic islands are generally different from those of continental islands/fragments and continents (Carlquist 1974).

## Heterostyly on Oceanic Islands

In earlier studies, it was often reported that heterostylous species were absent from oceanic island floras, e.g. Hawaii (Carlquist 1974) and Galapagos (McMullen 1987). Subsequently, Pailler *et al*. (1998*b*) considered that heterostyly may be absent or rare on oceanic islands. In general, it is very difficult to confirm whether heterostylous species are absent in particular islands because heterostyly is inconspicuous and often unnoticed in fields. In addition, the category of heterostyly or stylar polymorphisms has not been included in floristic surveys in many regions (e.g. Abe 2006; Tseng *et al*. 2008; Schlessman *et al*. 2014). Moreover, species with morphologically distylous flowers are sometimes functionally dioecious (e.g. *Mussaenda parviflora*, Naiki and Kato 1999; *Psychotria rubra*, Watanabe *et al*. 2014*b*). Several recent studies indicate that heterostyly may be more common on oceanic islands than indicated by previous studies. Although the presence of heterostylous species was not mentioned previously in the Galapagos Islands (McMullen 1987), two distylous species were recently reported: *Cordia lutea* (McMullen 2012) and *Waltheria ovata* (Bramow *et al*. 2013; Table 1). Similarly, two *Psychotria* species endemic to the Bonin Islands were found to be distylous: *P. boninensis* (Kondo *et al*. 2007; Sugawara *et al*. 2014) and *P. homalosperma* (Watanabe *et al*. 2014*a*). On La Reunion Island of the Mascarene Islands, distyly in *Gaertnera vaginata* (Pailler and Thompson 1997) and three *Erythroxylum* species (Pailler *et al*. 1998*b*), and tristyly in *Hugonia serrata* (Thompson *et al*. 1996; Meeus *et al*. 2011) have been reported. In the Canary Islands, Olesen *et al*. (2003) reported *Jasminum odoratissimum* as the only example of distyly in the islands.

**Table 1.** Reproductive studies on heterostylous species reported from major remote oceanic islands. BS, breeding system. ^1^According to Pailler (1997), 27 indigenous species (6 genera/5 families) in the Mascarene Islands possibly are heterostylous including 5 species shown in the table. Those species are: *Erythroxylum* 4 spp. (Erythroxylaceae); *Hugonia* 2 spp. (Linaceae); *Olax psittacorum* (Olacaceae); *Gaertnera* 14 spp., *Psathura* 3 spp., *Danais* 3 spp. (Rubiaceae). ^2^Although Baker (1953) reported pollen and stigma dimorphism in several *Limonium* species from the Canary Islands, there is no evidence that they are heterostyly. Olesen *et al*. (2003) also stated that *Jasminum* is the only one distylous example in the islands. Thus, we excluded them from the table. 1. Kondo *et al*. (2007), 2. Sugawara *et al*. (2014), 3. Watanabe *et al*. (2014*b*), 4. McMullen (2012), 5. Schofield (1989), 6. Bramow *et al*. (2013), 7. Pailler and Thompson (1997), 8. Pailler *et al*. (1998*b*), 9. Thompson *et al*. (1996), 10. Meeus *et al*. (2011), 11. Olesen *et al*. (2003), 12. Lewis (1975).

Region	Island group	Species	Family	BS	Literature
Pacific Ocean	Bonin Islands	*Psychotria boninensis*	Rubiaceae	Distyly	1, 2
		*P. homalosperma*	Rubiaceae	Distyly	3
	Galapagos Islands	*Cordia lutea*	Boraginaceae	Distyly	4
		*Waltheria ovate*	Sterculiaceae	Distyly	5, 6
Indian Ocean	La Réunion Island (Mascarene islands^1^)	*Gaertnera vaginata*	Rubiaceae	Distyly	7
		*Erythroxylum laurifolium*	Erythroxylaceae	Distyly	8
		*E. sideroxyloides*	Erythroxylaceae	Distyly	8
		*E. hypericifolium*	Erythroxylaceae	Distyly	8
		*Hugonia serrata*	Linaceae	Tristyly	9, 10
Atlantic Ocean	Canary Islands^2^	*Jasminum odoratissimum*	Oleaceae	Distyly	11
Pacific and Indian Ocean		*Pemphis acidula*	Lythraceae	Distyly	12

Several pantropical species occurring on islands are heterostylous. *Pemphis acidula*, a littoral woody plant of Lythraceae distributed widely on pantropical islands, is distylous at least on several Indian Ocean Islands (Lewis 1975). Lewis and Rao (1971) suggested that distyly in *P. acidula* had probably been derived from tristyly based on its floral features, although there is no evidence that the distyly evolved on the islands. Interestingly, *P. acidula* in Mauritius is monomorphic as a consequence of breakdown of distyly on the island (Lewis and Rao 1971). *Guettarda speciosa*, a littoral woody plant of Rubiaceae occurring on pantropical islands, also exhibits stylar dimorphism (T. Sugawara, unpubl. data). *Pemphis* (Murray 1986) and *Guettarda* (Nakanishi 1988) disperse their propagules via water flotation, and are widely distributed from eastern Africa to the Pacific. This may imply a high number of migrants among island groups, as in *Ipomoea* (Miryeganeh *et al*. 2014). If their seeds arrive on particular islands repeatedly, the colonization of heterostylous plants may be facilitated.

Few heterostylous species and few studies of their reproductive biology are known from oceanic islands, and thus heterostyly is probably rare in such areas, as suggested by Pailler *et al*. (1998*b*). However, there may be more heterostylous species as yet unrecognized as indicated by recent discoveries in the Bonin and Galapagos Islands. Further investigations will be necessary to ascertain the occurrence of heterostyly on oceanic islands.

## Breeding Systems of the Genus *Psychotria* in the Pacific Islands

*Psychotria* (Rubiaceae) is a large genus comprising more than 1800 woody species and occurs widely in tropical to subtropical regions of the world (Sohmer 1977; Hamilton 1990; Nepokroeff *et al*. 1999; Davis *et al*. 2001; 2009). Its ancestral breeding system is thought to be distyly (Hamilton 1990). This genus provides an excellent opportunity to study distyly on islands, because (i) it contains the largest number of distylous species (at least 127 spp. or more), (ii) it occurs frequently on remote oceanic islands, (iii) it has speciated on many islands and (iv) it contains examples of dioecism derived from distyly. In the Pacific islands, more than 400 species have been reported so far (Table 2).

**Table 2.** Species number of *Psychotria* in East Asia, Pacific Islands and neotropics. ^1^There are at least still several undescribed *Psychotria* species (I. Meyer, pers. comm.) in the Pacific Ocean, and some other genera (e.g. Hydnophytum, Amaracarpus, Dolianthus, Calycosia, Squamellaria) are thought to be included within the genus *Psychotria* (Barrabé *et al*. 2014; Razafimandimbison *et al*. 2014). On the other hand, ∼20 species from the Pacific will be transferred to the genus *Margaritopsis* sect. Palicoureeae (Barrabé *et al*. 2012). Thus the species number shown here is just an estimate. ^2^Estimated extinct species.

Region	Area	No. of species	Literature
Continental East Asia	China	16	Tao and Taylor (2011)
	French Indo-China	26	Pitard (1907)
Eastern Asia-Pacific Islands^2^	ML Japan	1	Yamazaki (1993)
	Bonin Islands (Japan)	2	Toyoda (2003)
	Ryukyu Islands (Japan)	3	Yamazaki (1993)
	Taiwan	4	Yang (1998)
	Philippines	95	Sohmer and Davis (2007)
	Irian Jaya	∼80 to 200	Sohmer (1988)
	Papua NG/Bismark	115	Sohmer (1988), Takeuchi (2007)
	Mariana Islands	4	Wagner *et al*. (2012) (Smithsonian website)
	Caroline Islands	15	Wagner *et al*. (2012) (Smithsonian website)
	Australia	16	Australian Plant Name Index (2015)
	New Zealand	0	Moore and Edgar (1970)
	New Caledonia	81	Barrabé *et al*. (2014), Barrabé (2014)
	Fiji	76	Smith (1988)
	Tonga	5	Yuncker (1959)
	Samoa	20	Whistler (1986)
	Marquesas Islands (FP)	13	Lorence and Wagner (2005), Wagner and Lorence (2002) (Smithsonian website)
	Society Islands (FP)	11	Meyer *et al*. (2003)
	Australes Islands (FP)	3	Meyer *et al*. (2003)
	Hawai'i Islands	11	Sohmer (1978), Wagner *et al*. (1999), Wagner *et al*. (2005) (Smithsonian website)
	Galapagos Islands	2	Tye and Francisco-Ortega (2011)
	Juan Fernandez Islands	0	Marticorena *et al*. (1998), Danton *et al*. (2006), Bernardello *et al*. (2006)
	Easter Island	1^2^	Orliac and Orliac (2008), Dubois *et al*. (2013)
Neotropics		208	Sedio *et al*. (2013)

If heterostyly is truly rare on oceanic islands as we hypothesized, most of these *Psychotria* species on oceanic islands in the Pacific may have evolved into other breeding systems from distyly before or after their colonization. The genus *Psychotria* is known as a ‘hyper-diversified woody genus’ in the tropics (Sedio *et al*. 2013). For example, only two colonists diversified into 81 species in New Caledonia (Barrabé *et al*. 2014), and a single colonist into 11 species in Hawaii (Table 2) (Nepokroeff *et al*. 2003). High species diversification is also found in the Philippines (95 species; Sohmer and Davis 2007), Fiji (76 species; Smith 1988), Papua New Guinea and the Bismarck Islands (115 species; Sohmer 1988), Marquesas (13 from three species; Lorence and Wagner 2005), etc. Despite the species diversity in each island, most species have never been studied from a reproductive perspective. According to Naiki (2012), *Psychotria* contains at least 127 distylous species, second only to *Primula* (Primulaceae) with 134 species in the number of species. Although only 127 out of 1800 *Psychotria* species are confirmed to be distylous, most of the other species yet to be studied are also considered to be distylous (Hamilton 1990). Thus the true number of distylous species in *Psychotria* should be much more, and probably this genus contains the most distylous species. Breakdown of distyly into monomorphy is frequently observed in the species occurring in the neotropics (Hamilton 1989, 1990; Sakai and Wright 2008), and evolution of dioecy from distyly has been also recognized in the Hawaiian *Psychotria* species (Sohmer 1977; Beach and Bawa 1980; Muenchow and Grebus 1989; Sakai *et al*. 1995*b*) and *P. rubra* in the Ryukyu Islands (Watanabe *et al*. 2014*b*).

Although genus-wide studies on reproductive biology of *Psychotria* have not been performed, the number of reproductive and molecular phylogeographic studies on *Psychotria* in the neotropics has increased during the past several decades (e.g. Hamilton 1989, 1990; Stone 1995, 1996; Almeida and Alves 2000; Faivre and McDade 2001; Castro and Oliveira 2002; Castro and Araujo 2004; Castro *et al*. 2004; Ramos and Santos 2006; Ramos *et al*. 2008; Sakai and Wright 2008; Paul *et al*. 2009). In Central and South America, most species of *Psychotria* maintain functional distyly, but some species are morphologically distylous and possess partial intramorph compatibility, and others are monomorphic (e.g. Bawa and Beach 1983; Hamilton 1989, 1990; Faivre and McDade 2001; Castro and Oliveira 2002; Castro and Araujo 2004; Castro *et al*. 2004; Coelho and Barbosa 2004, Rossi *et al*. 2005; Virillo *et al*. 2007; Sakai and Wright 2008; Souza *et al*. 2008; Consolaro *et al*. 2011; Faria *et al*. 2012). Several species, such as *P. brachiate*, *P. graciliflora*, *P. micrantha* (Sakai and Wright 2008), *P. carthagenensis* (Consolaro *et al*. 2011; Faria *et al*. 2012), show both distyly and monomorphy in different populations. However, no example of transition from distyly to dioecy has been discovered in the neotropics.

In contrast to neotropical species, only a few reproductive studies have been reported in other areas, including Africa (Baker 1958), Indonesia (Ernst 1932), Hawaii (Sohmer 1977) and East Asia (Kondo *et al*. 2007; Sugawara *et al*. 2013, 2014; Watanabe *et al.* 2014*a*, *b*, 2015). Hawaiian *Psychotria* is one of the well-known examples of dioecy derived from distyly (Vuilleumier 1967; Beach and Bawa 1980; Muenchow and Grebus 1989; Barrett 1992). In the Hawaiian Islands, all 11 *Psychotria* species form a monophyletic group (Nepokroeff *et al*. 2003), and it has been believed that they are probably derived from a distylous colonist (Beach and Bawa 1980; Sakai *et al*. 1995*b*), since distyly is basal for the genus (Sohmer 1978). This modification has contributed to a high proportion of dioecism and low proportion of distyly on oceanic islands. However, there has been no direct evidence that the single colonist of Hawaiian *Psychotria* is distylous, because the closest sister species and its breeding system are still unclear. Moreover, although both dioecism and distyly promote outcrossing, selection against selfing or inbreeding depression cannot be a driving force of this evolution (Bawa 1980). To understand the evolutionary pathway of dioecism in Hawaiian *Psychotria*, more precise investigations on reproductive system and phylogenetic relationship among the related species are required.

**Figure 1.**
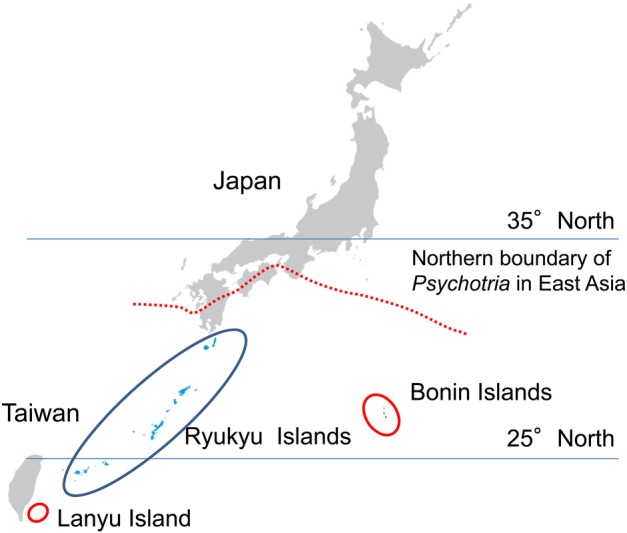
Distribution of *Psychotria* species in two subtropical island groups of Japan and Taiwan in East Asia. The Ryukyu Islands are continental, whereas the Bonin (Ogasawara) Islands and Lanyu (Orchid) Island are oceanic.

In the western Pacific, six *Psychotria* species occur on the Bonin (Ogasawara) Islands, the Ryukyu Islands and Lanyu (Orchid) Island. The volcanic oceanic Bonin Islands consist of ∼50 islands located ∼1000 km south of the Japanese mainland, while the continental Ryukyu Islands include ∼200 islands scattered between Taiwan and the Japanese mainland (Fig. 1). The Ryukyu Islands and Taiwan once were a part of the Chinese continent. Volcanic oceanic Lanyu Island is located off the southeastern coast of Taiwan (∼60 km), but it has never been connected to the mainland of Taiwan or other landmass (Fig. 1). Our preliminary phylogenetic study, following Razafimandimbison *et al*. (2014), suggested that six *Phychotria* species in the Bonin and the Ryukyu Islands and Lanyu Island are divided into three lineages (Fig. 4).

**Figure 2.**
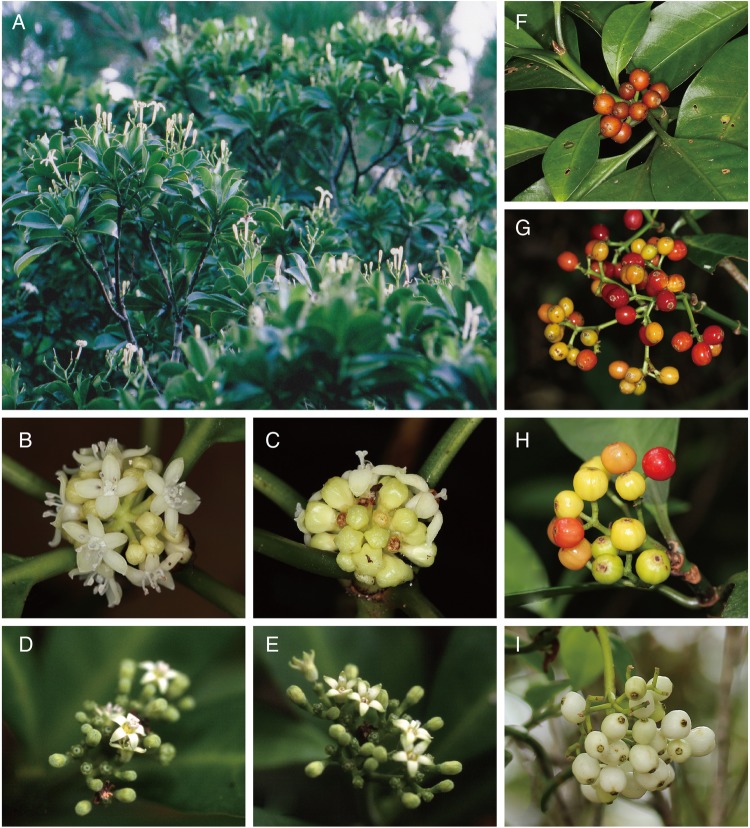
Inflorescences and fruit of six *Psychotria* species on the islands of Japan and Taiwan. Inflorescences and flowers (A) of *P. homalosperma* endemic to the Bonin Islands. Short- (B) and long-styled (C) flowers and fruit (F) of *P. cephalophora* on Lanyu Island. Short- (D) and long-styled (E) flowers of *P. boninensis* endemic to the Bonin Islands. Fruit (G) of *P. manillensis*, fruit (H) of *P. rubra* and fruit (I) of *P. serpens*, all in the Ryukyu Islands.

**Figure 3.**
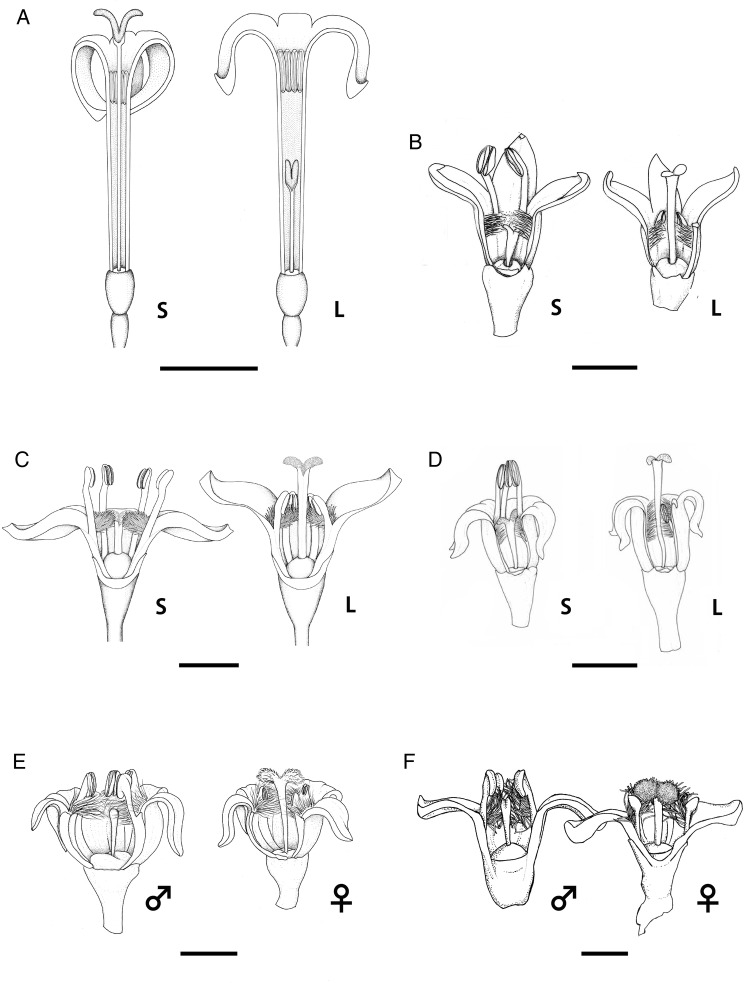
Flowers of six *Psychotria* species in Japan and Taiwan. Two flower morphs in distylous *P. homalosperma* (A), *P. boninensis* (B), *P. cephalophora* (C), *P. serpens* (D) and two flower morphs in dioecious *P. rubra* (E) and monoecious *P. manillensis* (F). S, short-styled flower; L, long-styled flower; ♂, male flower; ♀, female flower. Scale bars represent 5 mm in (A) and 2.5 mm in (B) to (F). Drawings are modified after Watanabe *et al*. (2014*a*) (A), Kondo *et al*. (2007) (B), Watanabe *et al*. (2015) (C), Sugawara *et al*. (2013) (D), Watanabe *et al*. (2014*b*) (E).

We expected that *Psychotria* species on the oceanic islands would have dioecy or self-fertility derived from distyly, while the species on the continental islands maintain functional distyly. However, the results from current field investigations are contradictory to our hypotheses; all three species on the oceanic island (*P. homalosperma* and *P. boninensis*, both endemic to the Bonin Islands, and *P. cephalophora* on the Lanyu Island; Fig. 1) are distylous, while the other three species in the continental Ryukyu Islands show various breeding systems including distyly (*P. serpens*), dioecy (*P. rubra*) and monoecy (*P. manillensis*; (Figs 2 and 3; Table 3). *Psychotria homalosperma* and *P. boninensis* are the first example of distyly on the oceanic Bonin Islands. Functional dioecy in *P. rubra* is the second example in the genus, following Hawaiian species. Monoecism in *P. manillensis* is a unique example, because there has been no report of monoecism in distylous species groups (Naiki 2012).

**Table 3.** Breeding systems of six *Psychotria* species occurring in Islands of Japan and Taiwan, and other examples of distylous species on remote oceanic islands. Oc, oceanic islands; Con, continental islands; S, short-styled; L, long-styled; Homo, homostyly; Herm, hermaphrodite; nd, no data. Ref., references; 1. Watanabe *et al*. (2014*b*), 2. Kondo *et al*. (2007), 3. Sugawara *et al*. (2014), 4. Sugawara *et al*. (2013), 5. Watanabe *et al*. (2014*a*), 6. Watanabe *et al*. (2015), 7. Mcmullen (2012), 8. Bramow *et al*. (2013), 9. Philipp *et al*. (2006), 10. Pailler and Thompson (1997), 11. Pailler *et al*. (1998*b*), 12. Thompson *et al*. (1996), 13. Meeus *et al*. (2011), 14. Olesen *et al*. (2003), 15. K. Watanabe and T. Sugawara (unpubl. data). **P < 0.001; *P < 0.01; NS, not significantly different = *P* > 0.05 after binomial test. ^1^Leaky (partially compatible, fruit/seed set or pollen tubes reached at the base of the style is more than 5%). ^2^Percentages of pollen tubes reached at the base of the style. ^3^All morphs were pooled. ^4^Seed set. ^5^Percentages of fruiting individuals out of 10 individuals examined.

Island group Species	Ref.	Breeding system	Morphs	No. of pop. examined	Morph		Compatibility		Fruit set after hand pollination (%)						Pollination		
									Intermorphic		Self-		Intramorphic		Open fruit set (%)		Pollinator
					Morph ratio (S/L)		SI/SC	Intramorphic	L ⇒ S	S ⇒ L	S-self	L-self	S–S	L–L	L	S	
Bonin Islands (Oc)																	
*P. homalosperma*	1, 15	Distyly	S, L	3	1.5–2.3	**	SI	LL, SS^1^	82.4	82.6	0	0	5.9	0	2.8–8.9	0.2–1.4	Bee (moth)
*P. boninensis*	2, 3	Distyly	S, L	1	1.05	NS	SI	LL, SS	7.1	27.6	0	0	0	0	12.6; 18.6	9.2; 8.4	Bee (bee)
Ryukyu Islands (Con)																	
*P. serpens*	4, 15	Distyly	S, L, Homo	3	nd	–	SI	LL, SS^1^	78.9	85.0	0	0	6.3	0	53.4	40.2	Bee, Wasp
*P. rubra*	5	Dioecy	Male, Female	4	1.3–2.0	**	–	–	0	64.9	–	–	–	–	40.5	0	Fly, Wasp
*P. manillensis*	15	Monoecy	Herm, Female	5	–	–	SC	–	–	–	–	–	–	–	11.9	N	Fly, Wasp
Lanyu Island (Oc)																	
*P. cephalophora*	6	Distyly	S, L	1	1.0	NS	SI	LL^1^, SS	100^2^	100^2^	0^2^	3.3^2^	0^2^	5^2^	nd	nd	Moth, beetle?
Galapagos (Oc)																	
*C. lutea*	7	Distyly	S, L	1	nd	–	SC^1^	LL^1^, SS	74^3^		25^3^		9^3^		20^3^		Beetle, bee
*W. ovate*	8, 9	Distyly	S, L	12	1.33–0.56		SC^1^	LL, SS^1^	68^4^	61^4^	20^5^	40^5^	21^4^	4^4^	54^4^	75^4^	Bee, fly, grasshopper
La Reunion (Oc)																	
*G. vaginata*	10	Distyly	S, L	18	0.75–1.82	*	SI	LL, SS^1^	92.2	92.8	0	0	19.4	0	29–49	21–39	nd
*E. laurifolium*	11	Distyly	S, L	3	0.78–1.44	NS	SI	LL^1^, SS	72.4	69.8	1.6	3.1	2.0	12.8	nd	nd	Moth?
*E. sideroxyloides*	11	Distyly	S, L	1			nd	nd	nd	nd	nd	nd	nd	nd	nd	nd	nd
*E. hypericifolium*	11	Distyly	S, L	1			nd	nd	nd	nd	nd	nd	nd	nd	nd	nd	nd
*H. serrata*	12, 13	Tristyly	S, M, L	1	1S: 3M: 5L	–	SI	nd	66.7^3,4^		3.6^3,4^		nd	nd	S: 27^4^, M: 43^4^, L: 44^4^		Bee (butterfly?)
Canary Islands (Oc)																	
*J. odoratissimum*	14	Distyly	S, L	2	0.91; 1.54	NS	nd	nd	nd	nd	nd	nd	nd	nd	nd	nd	nd

Why has distyly been maintained or not on the islands, and dioecy/monoecy have been evolved? And now, we present some characteristics of the breeding system for several species of *Psychotria* obtained from our studies.

*Psychotria homalosperma*, an endemic and endangered tree (up to 12 m), is distylous with self-incompatibility (Watanabe *et al*. 2014*a*). Its flowers are white, long and narrow tubular with strong floral scent that contains large proportion of linalool (K. Watanabe, unpubl. data). Based on these features, we suppose that this species is adapted to hawkmoth pollinators. In the Bonin Islands, however, anthropogenic disruption of the insect fauna has been reported (Kato *et al*. 1999; Abe 2006; Abe *et al*. 2008). In our field investigations, the main flower visitor is an introduced honeybee (*Apis mellifera*), and moth visitation was recorded only once in more than 100 h of observations (Watanabe *et al*. 2014*a*; K. Watanabe, unpubl. data). This fact suggests that the shift of pollinators from long- to short-tongued insects, caused by human activity, may have occurred in this species on the oceanic Bonin Islands. This pollinator shift would result in unidirectional pollen flow from the S- to L-morphs (Fig. 5). In fact, the L-morph sets fruit 1.7–38 times more than the S-morph in the field (K. Watanabe, unpubl. data). This difference is so large that it cannot be explained solely by S-biased sex ratios (1.5–3.5) in the field (Watanabe *et al*. 2014*a*). If this situation continues, the S-morph would supply only pollen grains like a male plant and the L-morph would receive them like a female plant. Eventually, *P. homalosperma* may evolve into dioecy under this situation. However, *P. homalosperma* has not regenerated recently, probably because of seed predation by introduced rats and deforestation (Watanabe *et al*. 2009).

**Figure 4.**
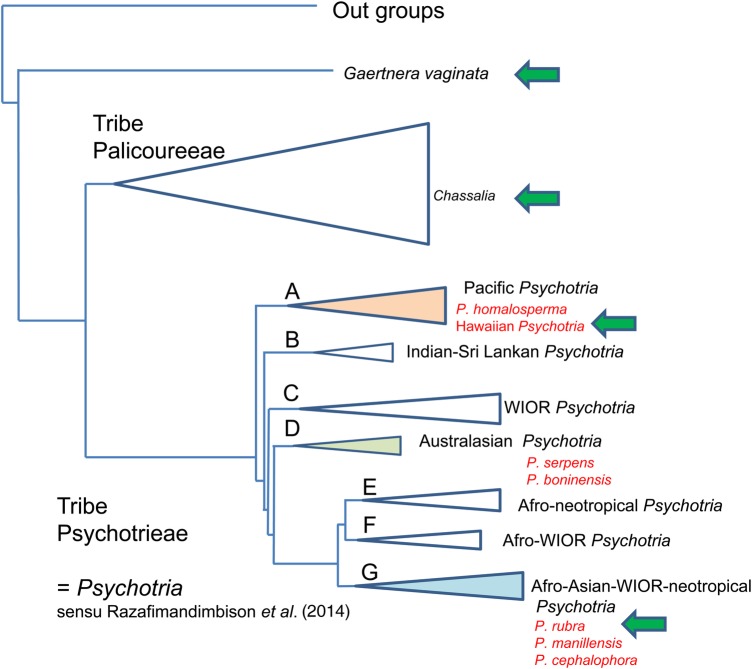
Phylogenetic relationships and breeding systems in *Psychotria* and its sister groups, including six species distributed in Japan and Taiwan. The phylogenetic hypothesis is modified from Razafimandimbison *et al*. (2014). Green arrows indicate the occurrence of the species in which dioecy evolved from distyly. WIOR, western Indian Ocean region.

*Psychotria serpens*, a woody climber, is basically distylous in the Ryukyu Islands, but the short homostylous morph, together with the S- and L- morphs, is occasionally found in a population on Okinawa Islands (Sugawara *et al*. 2013). According to Sugawara *et al*. (2013), this homostyly is a consequence of unusual development of filaments and stamen. Therefore, the homostylous plants occasionally found on the Okinawa Island may be exceptional for the species. According to the molecular phylogenetic data (Fig. 4), *P. boninensis*, a woody climber endemic to the Bonin Islands, is supposed to be derived from *P. serpens.* Thus the ancestral colonist of *P. boninensis* on the Bonin Islands also appears to be distylous.

*Psychotria rubra*, a shrub of non-limestone broad-leaved forests in the Ryukyu Islands is morphologically distylous with L- and S-morphs (Watanabe *et al*. 2014*b*). However, the S-morph never set any fruit, whereas the L-morph never had pollen grains (Watanabe *et al*. 2014*b*). Therefore, this species is functionally dioecious. Considering the morphological features, the dioecy found in *P. rubra* is probably derived from distyly. To discuss the evolutionary pathway of dioecy in *P. rubra*, we need to compare precise reproductive systems among the closely related species with a reliable phylogenetic tree.

*Psychotria manillensis*, a shrub of limestone broad-leaved forests in the Ryukyu Islands, is monoecious and self-compatible (Table 3) (K. Watanabe, unpubl. data). This reproductive system is particularly interesting, because no other monoecism has been found in other distylous species groups in flowering plants. Theoretically, it is very difficult to explain the evolution of monoecism from distyly because distyly is a genetically controlled dimorphism. Although the detailed phylogenetic relationships among the species are still unsolved, *P. manillensis* is closely allied to *P. rubra* based on morphology and preliminary molecular data (Fig. 4). Cytologically, *P. manillensis* is octoploid (2*n* = 84), while *P. rubra* is tetraploid (2*n* = 42) (Nakamura *et al*. 2003). These chromosomal data suggest that *P. manillensis* may be a polyploid derivative of *P. rubra*. If the genes responsible for sexual determination of flowers became heterogeneous through doubling of chromosomes, the plant can have both male and female flowers within a single plant (monoecy).

## Reproduction and Evolutionary Modification of Heterostyly on Islands

What makes heterostyly rare in floras of isolated islands? Three possible reasons can be raised for its rarity on islands: (i) failure of colonization, (ii) difficulty in autochthonous evolution of distyly and (iii) the evolutionary modification into other breeding systems such as self-compatibility, dioecism, monoecism.

Failure of colonization should be mostly due to self-incompatibility of heterostylous species (Pailler *et al*. 1998*b*). It is surely difficult for self-incompatible species to reproduce on islands compared with self-compatible species, as mentioned by Baker (1955). However, the rarity of heterostyly on islands may not be explained only by this reason, because many dioecious colonists are known from oceanic islands. In fact, almost one-third of the extant dioecious species in the Hawaiian Islands are derived from dioecious ancestors (Sakai *et al*. 1995*b*). Although heterostyly is much less common than dioecy even in mainland areas, some heterostylous plants may reach the remote oceanic islands.

Colonization of oceanic islands by reproductively dimorphic plants is often associated with bird dispersal (Bawa 1980; Lloyd 1985; Sakai *et al*. 1995*a*; Webb *et al*. 1999). One of the reasons for this may be because clumped dispersal of propagules facilitates colonization of dimorphic plants (Sakai *et al*. 1995*a*). Of 11 heterostylous species that occur on oceanic islands (Table 1), 9 (*Cordia*, *Erythroxyluym*, *Gaertnera*, *Hugonia, Jasminum* and *Psychotria*) have fleshy fruits, and at least four of them have seeds dispersed by birds (*Psychotria,* Ono and Sugawara 1981; *Cordia*, Heleno *et al*. 2011; *Gaertnera*, Malcomber 2002). Their colonists are presumably dispersed by birds, like dioecious colonists in other oceanic islands. On the other hand, *Waltheria* (Carlquist 1974), *Pemphis* (Murray 1986) and *Guettarda* (Nakanishi 1988) have sea-drifted seeds, and this mode of dispersal may also be related to their colonization.

Of 11 heterostylous species occurring on oceanic islands (Table 1), six species (*P. homalosperma*, *P. boninensis*, *P. cephalophora*, *Gaertnera vafinata*, *Erythroxylum laurifolim* and *H. serrata*) are basically self-incompatibile (Table 3), although *P. cephalophora* and *E. laurifolium* are slightly self-compatible. This fact suggests that multiple ancestors may have colonized the islands. *Cordia* and *Waltheria* in the Galapagos Islands show partial self-compatibility, known as ‘leaky incompatibility’ (McMullen 2012; Bramow *et al*. 2013). This leaky self-incompatibility might help their initial colonization and spread among islands in the Galapagos. It is notable that all 11 species are long-lived woods, and their colonists might survive long enough for conspecifics to colonize (Meeus *et al*. 2011).

Because heterostylous plants require pollinators suitable for their reproduction, the heterostylous species on oceanic islands provide evidence that continuous pollinator services exist on islands. This stable pollinator services might be unusual for oceanic islands, where ecosystems are usually unstable (Abe 2006).

Geography is also important for understanding colonization and reproduction of heterostylous species on oceanic islands. Watanabe *et al*. (2015) reported distyly in *P. cephalophora* from oceanic Lanyu Island, which is only 60 km away from the south of Taiwan, but has never been connected to it. There seems to be several more distylous plant species on the island (K. Watanabe, pers. obs.). Many floristic elements are shared between Lanyu Island and other northern islands in the Philippines where *P. cephalophora* occurs (Nakamura *et al*. 2014). *Psychotria cephalophora* might have colonized Lanyu Island repeatedly, since it is located near the big continental islands, unlike more remote oceanic islands. Moreover, ∼700 native plant species occur on Lanyu Island (50 km^2^ in area) (Tseng *et al*. 2008), which is more than twice as many species as in the Bonin Islands (∼70 km^2^) (Toyoda 2003). This environment with rich species probably supports the reproduction and maintenance of heterostylous species on oceanic Lanyu Island.

Some remote oceanic islands, like the Hawaiian and the Bonin Islands (5000 and 1000 km from the nearest continent or continental islands, respectively), are quite isolated from any other landmass, while some other oceanic islands close to the mainland, like Lanyu Island and Izu-Oshima Islands (20 km from mainland Japan), are largely affected by continental biota. We need to take these geographical and historical conditions into account to understand how heterostylous species colonize and reproduce on oceanic islands.

The rarity of heterostyly on islands may also result from the difficulty in autochthonous evolution of heterostyly on islands. Outcrossing is advantageous for island plants, and it may drive monomorphic plants to evolve dioecy within islands (*in situ* evolution) because dioecy is easily established (Baker and Cox 1984). Indeed, there are many examples of *in situ* evolution of dioecism on islands (e.g. Sakai *et al*. 1995*b*; Baker and Cox 1984). Compared with establishment of dioecy, however, the establishment of heterostyly is far more difficult (Carlquist 1974), because of the complexity of the genetic control of heterostyly (Lewis and Jones 1992).

The paucity and bias of pollinator fauna and small population sizes on islands may cause the evolutionary modification of heterostyly into other breeding systems. The breakdown of heterostyly into monomorphy can be caused by recombination within ‘a heterostylous gene’ (Charlesworth and Charlesworth 1979*a*; Washitani 1996), but this recombinant cannot spread in natural populations under a stable environment because distyly is an efficient mechanism to avoid inbreeding depression (Charlesworth and Charlesworth 1979*a*). However, the recombinants would spread when suitable pollinators decline or the population size is decreased (Washitani *et al*. 1994; Washitani 1996).

Low availability of pollinators and small plant population sizes are the characteristic features on oceanic islands. Thus, the evolutionary breakdown of heterostyly can be caused more frequently on oceanic islands than on continents, which may contribute to the rarity of heterostyly on islands. Recently, Lorence and Wagner (2005) reported that flowers of many *Psychotria* species in the Marquesas appeared to be homostylous. Of course more precise observations and experiments are needed to confirm their functional breeding systems and evolutionary pathways, but there may be a number of *Psychotria* species having undetected homostyly in the Pacific.

In the Caribbean Islands, although they are not oceanic islands but continental fragments, several interesting evolutionary modifications of heterostyly have been reported (reviewed in Barrett *et al*. 1996). Self-compatible, tristylous *Eichhornia paniculata* lost one of its morphs on Jamaica, probably because of poor pollinator services (Barrett *et al*. 1996). A similar example was reported from *Turunera ulmifolia*, of which more self-compatible homostylous varieties and fewer self-incompatible distylous varieties were found on small islands in contrast to continents (Barrett and Shore 1987).

Evolution of dioecy from distyly is found much less frequently than the evolution of self-fertilization is (Barrett and Richards 1990). Muenchow and Grebus (1989) listed eight genera as possible examples of this pattern. We revised this list in Table 4 based on the recent literature; 3 of 10 genera listed in Table 4 may have occurred on islands.

**Table 4.** Examples of the plants evolved from distyly to dioecy. ^1^Including dioecious genera within genus *Guettarda*.

Family	Genus	Location	Literature
Rubiaceae	*Chassalia*	La Reunion	Pailler *et al*. (1998*a*)
	*Coussarea*	Costa Rica	Beach and Bawa (1980)
	*Gaertnera*	Paleotropics	Van Beusekom (1967), Malcomber (2002), Malcomber and Taylor (2009)
	*Guettarda* (*Timonius*/*Antirhea*)^1^	Worldwide	Achille *et al*. (2006) (*Guettarda*), Litrico *et al*. (2005) (*Antirhea*)
	*Mussaenda*	Asia	Baker (1958), Naiki and Kato (1999)
	*Psychotria*	Hawaiian Islands Ryukyu Islands	Vuilleumier (1967), Sohmer (1977), Hamilton (1990) Watanabe *et al*. (2014*b*)
	*Phyllopentas*	Madagascar	Andriamihajarivo *et al*. (2011)
Boraginaceae	*Cordia*	Costa Rica	Opler *et al*. (1975)
Menyanthaceae	*Nymphoides*	North America	Ornduff (1966)
Elythroxylaceae	*Erythroxylum*	Costa Rica	Bawa and Opler (1975), Ganders (1979*a*), Abarca *et al*. (2008)

**Figure 5.**
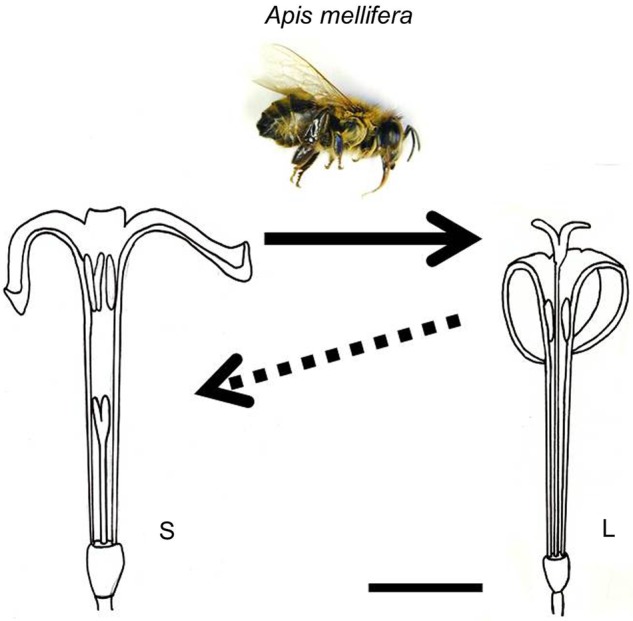
Introduced honeybees (*A. mellifera*) frequently visit flowers of *P. homalosperma* to collect pollen grains in the oceanic Bonin (Ogasawara) Islands. They transport pollen grains only towards stigmas of the long-styled morph, and thus would result in unidirectional pollen flow from the short- to long-styled flowers. Arrows indicate pollen flows. Scale bar = 5 mm.

Several hypotheses may account for the evolution from distyly to dioecy (e.g. Ornduff 1966; Beach and Bawa 1980; Webb 1999; Rosas and Domínguez 2009). Beach and Bawa (1980), following Robertson (1892) and Baker (1958), proposed that the unidirectional (one-way) pollen flow from S- to L-morphs leads to separate sexes. A pollinator shift from long- to short-tongued pollinators initiates unidirectional pollen flow from S- to L-morphs (Fig. 5), resulting in the L-morph becoming male-sterile (Beach and Bawa 1980). It eventually drives the evolution of the breeding system from distyly to dioecy. Muenchow and Grebus (1989) suggested that the complete disappearance of the long-tongued pollinators at the initial stage and the male sterility of the L-morph were required for the scenario. A change of pollinators could occur on oceanic islands too, since the pollinator fauna is usually poorer on oceanic islands than on continents (Barrett *et al*. 1996). For example, a distylous plant whose original pollinator is a long-tongued hawkmoth would experience a complete pollinator shift from long- to short-tongued pollinators following colonization of an island where only short-tongued insects are available. *Psychotria homalosperma*, which has functional distyly on the Bonin Islands, may show the initial stage of this transition.

Polyploidization and hybridization are assumed to have played an important role in the evolution of several taxa in the Hawaiian Islands (reviewed in Keeley and Funk 2011). These phenomena may have also driven the evolution of breeding systems on other islands. Recently, a correlation between breakdown of distyly and polyploidy has been recognized in several taxa (*Damnacanthus*, Naiki and Nagamasu 2004; *Turnera*, Shore *et al*. 2006; *Ophiorrhiza*, Nakamura *et al*. 2007). Later, Naiki (2012) concluded in his review of a wide range of taxa across the world that polyploidy was not always accompanied with the breakdown of distyly, but that following the breakdown of distyly, polyploidy in monomorphic plants could decrease the expression of inbreeding depression. Although the process of evolution of monoecy in octoploid *Psychotria manillensis* still remains unclear, genetic recombination or chromosomal doubling may be involved in the evolution of its unusual breeding system.

## Future Prospects

Heterostyly on remote islands provides an opportunity to deepen our understanding of plant reproductive biology. We propose the following three steps in future investigations. First, we need comparative studies within lineages to investigate breeding systems of island species and their closest continental relatives. Although few heterostylous lineages occur on islands, genera such as *Psychotria* provide excellent opportunities for analysis of evolutionary modifications of heterostyly associated with colonization of oceanic islands. To understand the reproductive system of each species, a study should include not only its floral traits, but also its functional breeding system, incompatibility, open fruit set, plant–pollinator interactions and pollen flow. Second, we need to construct a robust phylogenetic framework to trace the evolutionary pathway of each species with high confidence. Third, we also need to consider the environmental and historical background of islands. The simple classification into ‘oceanic’ or ‘continental’ islands might not be enough to explain the ecological background. Instead, careful examinations of correlations between breeding systems and geographical/historical conditions are needed to understand the general patterns of heterostyly on islands.

## Sources of Funding

This study was partly supported by JSPS KAKENHI Grant (no. 26840130 to K.W., no. 22570096 to T.S.) and the Environment Research and Technology Development Fund (4-1402) of the Ministry of the Environment, Japan.

## Contributions by the Authors

K.W. and T.S. contributed to writing of the paper.

## Conflict of Interest Statement

None declared.
